# c-MYC-induced long noncoding RNA MEG3 aggravates kidney ischemia–reperfusion injury through activating mitophagy by upregulation of RTKN to trigger the Wnt/β-catenin pathway

**DOI:** 10.1038/s41419-021-03466-5

**Published:** 2021-02-18

**Authors:** Dajun Liu, Ying Liu, Xiaotong Zheng, Naiquan Liu

**Affiliations:** Department of Nephrology, Shengjing Hospital of China Medical University, 110022 Shenyang, Liaoning China

**Keywords:** Apoptosis, Long non-coding RNAs

## Abstract

Ischemia–reperfusion injury (IRI)-induced acute kidney injury (AKI) is a life-threatening disease. The activation of mitophagy was previously identified to play an important role in IRI. Maternally expressed 3 (MEG3) can promote cerebral IRI and hepatic IRI. The present study was designed to study the role of MEG3 in renal IRI. Renal IRI mice models were established, and HK-2 cells were used to construct the in vitro models of IRI. Hematoxylin–eosin staining assay was applied to reveal IRI-triggered tubular injury. MitoTracker Green FM staining and an ALP kit were employed for detection of mitophagy. TdT-mediated dUTP-biotin nick-end labeling assay was used to reveal cell apoptosis. The results showed that renal cortex of IRI mice contained higher expression of MEG3 than that of sham mice. MEG3 expression was also elevated in HK-2 cells following IRI, suggesting that MEG3 might participate in the development of IRI. Moreover, downregulation of MEG3 inhibited the apoptosis of HK-2 cells after IRI. Mitophagy was activated by IRI, and the inhibition of MEG3 can restore mitophagy activity in IRI-treated HK-2 cells. Mechanistically, we found that MEG3 can bind with miR-145-5p in IRI-treated cells. In addition, rhotekin (RTKN) was verified to serve as a target of miR-145-5p. MEG3 upregulated RTKN expression by binding with miR-145-5p. Further, MEG3 activated the Wnt/β-catenin pathway by upregulation of RTKN. The downstream effector of Wnt/β-catenin pathway, c-MYC, served as the transcription factor to activate MEG3. In conclusion, the positive feedback loop of MEG3/miR-145-5p/RTKN/Wnt/β-catenin/c-MYC promotes renal IRI by activating mitophagy and inducing apoptosis, which might offer a new insight into the therapeutic methods for renal IRI in the future.

## Introduction

Ischemia–reperfusion injury (IRI) is caused by a sudden temporary impairment of the blood flow to the particular organ and can cause a variety of ischemic diseases, including brain stroke, myocardial infarction, and ischemic acute kidney injury (AKI)^1^. Although reperfusion is essential for the survival of ischemic kidney tissues, it can lead to additional damage to the kidney that results in poorer prognosis of AKI patients^2,3^. AKI is characterized by tubular epithelial cell death and dysfunction at the cellular level^4^. The morbidity and mortality rates of patients with AKI in intensive care unit can reach 50–70%^5^.

Although the molecular mechanisms underlying apoptosis of renal tubular epithelial cells during IRI remain unclear, numerous studies indicate that mitochondrial dysfunction plays a critical role in apoptosis of tubular epithelial cells^6^. Cellular stresses, including hypoxia, ischemia, and glucose deprivation, can cause damage or dysfunction of mitochondria^7^. In addition, researches showed that mitophagy exerts a protective function in the liver^8^. Mitophagy, a complex process that rapidly and selectively removes long-lived or damaged mitochondria in an autophagy-dependent manner, is an adaptive response of mitochondria to various stress damages, which was previously identified to play an important role in IRI^9^. The most recognized mitophagy pathway in mammalian cells is dependent on Parkin^10^. A study revealed that Parkin-related mitophagy promotes cardiomyocyte death in IRI^11^. Another study has indicated that deficiency of autophagy aggravates tubular injury in proximal tubules and leads to damage of mitochondria in IRI-induced AKI model^12^.

Long noncoding RNAs (lncRNAs) are noncoding RNAs with the length >200 nucleotides and lack the protein-coding capacity^13^. LncRNAs are potential biomarkers or therapeutic targets for AKI^14^. The competing endogenous RNA (ceRNA) pattern refers to that lncRNAs competitively bind with microRNAs (miRNAs) to antagonize the suppressive effects of miRNAs on downstream targets^15^, and lncRNAs were widely reported to serve as ceRNAs in the pathogenesis of AKI. For instance, XIST binds with miR-142-5p to upregulate PDCD4 and thus alleviates AKI^16^. The LINC00343/rno-miR-1956-5p/KCP axis is involved in contrast-induced AKI^17^. LINC00520 upregulates OSMR expression to promote AKI progression by binding with miR-27b-3p^18^. TCONS_00016233 contributes to sepsis-induced AKI via modulation on the miR-22-3p/AIFM1 axis^19^. Moreover, lncRNAs are crucial mediators for mitophagy. PVT1 is activated during muscle atrophy and impacts mitochondrial respiration, mitophagy, and apoptosis in vivo^20^. RMST enhances FUS SUMOylation to inhibit mitophagy of glioma cells^21^.

Maternally expressed 3 (MEG3) is an imprinted gene with a location at 14q32.2 and is a significant mediator for IRI. MEG3 facilitates cerebral IRI by increasing pyroptosis via the miR-485/AIM2 axis^22^ and the Wnt/β-catenin pathway^23^. MEG3 contributes to hepatic IRI via the miR-34a/Nrf2 signaling pathway^24^. Moreover, MEG3 promotes myocardial IRI by downregulation of miR-7-5p^25^. MEG3 functions as a ceRNA against miR-21 to regulate ischemic neuronal death^26^. MEG3 inhibits cancer cell proliferation and acts as an antioncogene in gastric cancer^27^, prostate cancer^28^, and laryngeal cancer^29^. The functions and underlying mechanisms of MEG3 in the progression of kidney IRI remain to be further explored. We hypothesized that MEG3 can promote kidney IRI via the ceRNA pattern. In the present study, human kidney proximal tubular cells (HK-2) were used to establish an IRI model in vitro. Kidney IRI mice were used as the in vivo models. MEG3 was knocked down in IRI mice and in HK-2 cells, and loss-of-function assays revealed the effects of MEG3 on renal function and on cell apoptosis and mitochondrial dysfunction during IRI.

## Results

### MEG3 exhibited high expression after IRI

**Fig. 1:**
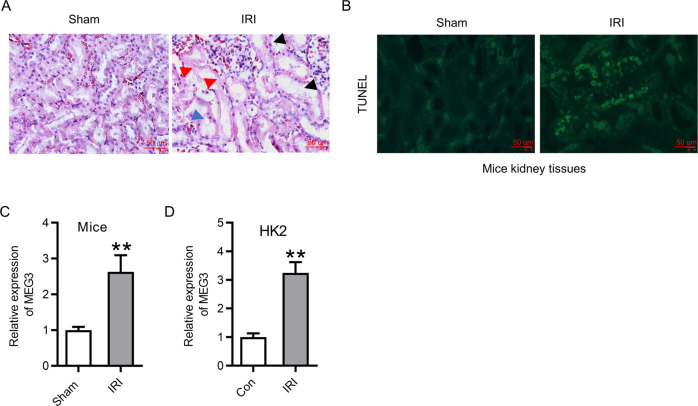
MEG3 exhibited high expression after IRI. **A** H&E staining revealed IR triggered tubular injury. **B** TUNEL assay revealed the apoptosis cells induced by IRI in mice kidney tissues. Red arrow indicates coagulative necrosis with tubular structures; black arrow indicates renal tubule vacuole; blue arrow indicates renal tubular necrosis and detachment. **C** RT-qPCR revealed expression of MEG3 over a 12-h post IRI in renal cortex of mice compared with that in the sham group. **D** RT-qPCR revealed the expression of MEG3 in HK-2 cells 6 h following IRI. **p < 0.01.

To identify the role of MEG3 in kidney IRI, we constructed IRI mice models. Results of hematoxylin–eosin (H&E) staining assay revealed that IRI triggered tubular injury (Fig. 1A). Moreover, a TdT-mediated dUTP-biotin nick-end labeling (TUNEL) assay revealed that IRI induced more apoptosis in kidney tissues of mice (Fig. 1B). These data illustrated that the IRI models were successfully established. Then reverse transcription quantitative real-time PCR (RT-qPCR) was conducted to evaluate the expression of MEG3 in renal cortex. The results revealed that MEG3 exhibited higher expression post IRI in renal cortex compared with that in the sham group (Fig. 1C). Similarly, the expression of MEG3 was elevated in HK-2 cells 6 h following IRI (Fig. 1D).

### Suppression of MEG3 inhibited apoptosis and mitophagy in HK-2 cells following IRI

**Fig. 2:**
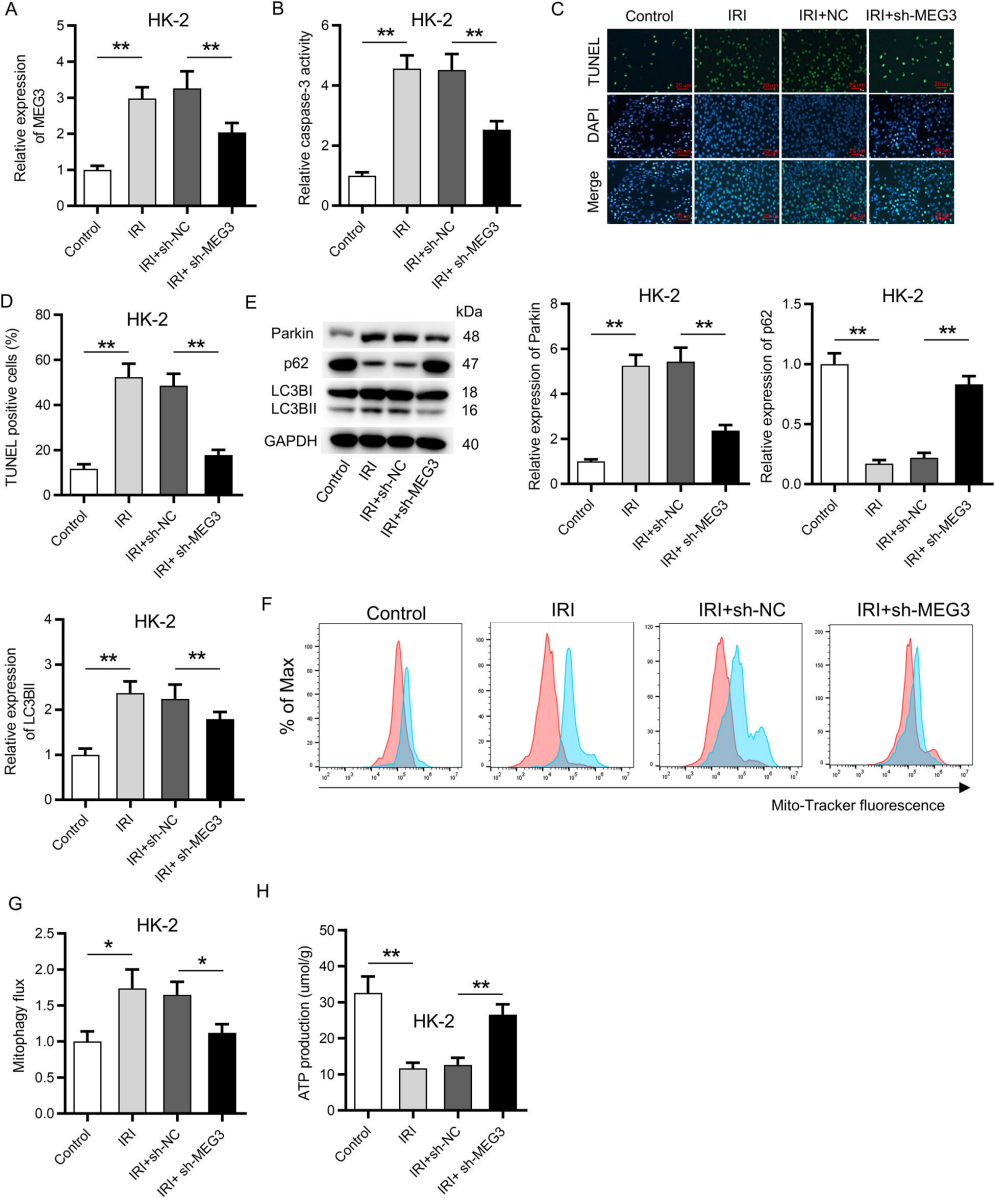
Suppression of MEG3 inhibited apoptosis and mitophagy in vitro. **A** RT-qPCR showed the transfection efficiency of sh-MEG3 in HK-2 cells. **B** A caspase-3 kit was applied to detect the activity of caspase-3 in sh-MEG3 transfected HK-2 cells. **C**, **D** A TUNEL assay revealed the influence of MEG3 deficiency on apoptosis of HK-2 cells. **E** Western blot analysis indicated the protein levels of Parkin, p62, LC3B-I, and LC3B-II. **F**, **G** MitoTracker Green FM staining was used to evaluate mitophagy flux under the influence of MEG3 deficiency. Red color indicated DMSO and blue color indicated Baf. **H** The influence of MEG3 deficiency on ATP production was determined via an XFe96 extracellular flux analyzer. *p < 0.05, **p < 0.01.

Furthermore, we intended to identify whether MEG3 is involved in the pathogenesis of IRI. MEG3 was knocked down in HK-2 cells by transfection of sh-MEG3. RT-qPCR analysis showed that IRI resulted in a significant increase of the expression of MEG3, and transfection of sh-MEG3 caused a significant decrease of the expression of MEG3 in HK-2 cells (Fig. 2A). Afterwards, activity of the caspase-3 (cell apoptosis index) was measured via a caspase-3 detection kit. The results showed that IRI enhanced the activity of caspase-3, and this effect was rescued by downregulation of MEG3 (Fig. 2B). Moreover, the increased number of TUNEL-positive cells caused by IRI was inversely changed by deficiency of MEG3 in HK-2 cells (Fig. 2C, D). These data demonstrated that downregulation of MEG3 inhibited the apoptosis of HK-2 cells after IRI. Thereafter, whether MEG3 is involved in mitophagy in HK-2 cells was explored. Protein levels of Parkin, LC3B-I, LC3B-II (an autophagosome marker), and p62 were detected. p62, a mitophagy adaptor, can translocate to damaged mitochondria and be digested during mitophagy, and the decrease of p62 indicates mitophagy^30^. Results from western blot analysis indicated that the protein levels of Parkin, LC3B-I, and LC3B-II were increased and the protein levels of p62 were reduced following IRI, while silencing of MEG3 rescued these effects (Fig. 2E). Subsequently, mitophagy flux was detected by measurement on the changes of mitochondria repressed by Baf (lysosome function inhibitor). As shown in Fig. 2F, G, mitophagy flux was significantly enhanced following IRI and was further reduced by silenced MEG3. In addition, ATP production was inhibited in response to IRI, while downregulation of MEG3 effectively neutralized this effect (Fig. 2H).

### Silencing of MEG3 attenuated the kidney injury in vivo

**Fig. 3:**
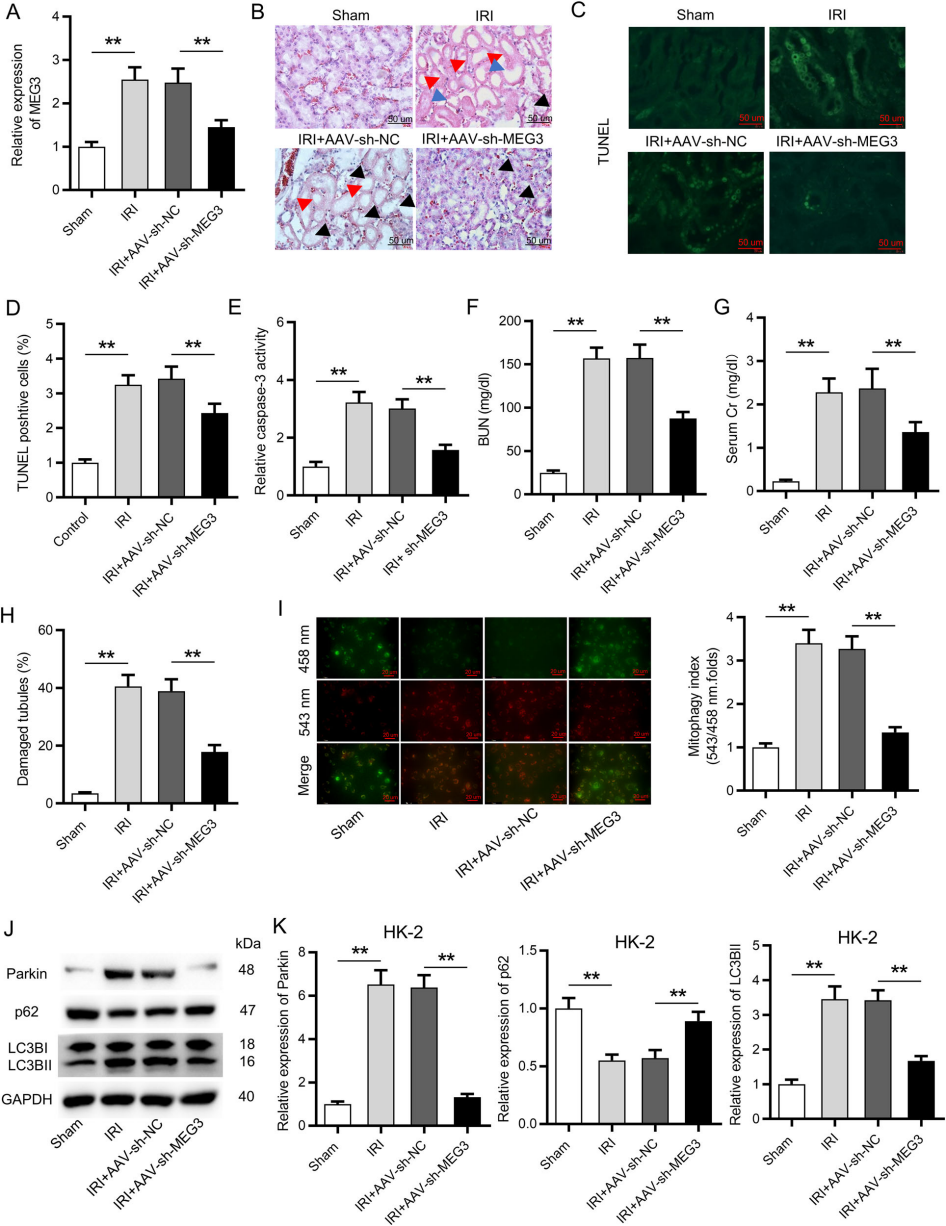
Silencing of MEG3 attenuated the kidney injury in vivo. **A** RT-qPCR indicated the expression of MEG3 in the kidney of mice after injection of AAV-sh-MEG3 vector. **B** H&E staining exhibited the effects of downregulation of MEG3 on tubular injury. Red arrow indicates coagulative necrosis with tubular structures; black arrow indicates renal tubule vacuole; blue arrow indicates renal tubular necrosis and detachment. **C**, **D** A TUNEL assay was applied to detect the number of TUNEL-positive cells. **E** Influence of silenced MEG3 on caspase 3 activity. **F**, **G** The effects of silenced MEG3 on the levels of BUN and creatinine in the serum were measured. **H** The effects of silenced MEG3 on tubular injury index were determined. **I** An mt-Keima assay was used to detect the mitophagy activity following IRI and under the influence of MEG3 deficiency. **J**, **K** Western blot analysis of Parkin, p62, LC3B-I, and LC3B-II levels in kidney tissues from IRI mice and sham mice. **p < 0.01.

Next, the effects of MEG3 suppression on kidney injury in vivo were investigated. RT-qPCR indicated that injection of AAV-sh-MEG3 vector led to a significant decrease of the expression of MEG3 in the kidney of mice (Fig. 3A). H&E staining assay exhibited that IR-triggered tubular injury was alleviated by downregulation of MEG3 (Fig. 3B). The increased number of TUNEL-positive apoptotic cells induced by IRI was decreased by MEG3 inhibition (Fig. 3C, D). Silencing of MEG3 rescued the increase of caspase-3 activity in renal tissues of IRI mice (Fig. 3E). Additionally, IR induced elevation of the levels of blood urea nitrogen (BUN) and creatinine in the serum were rescued by the suppression of MEG3 (Fig. 3F, G). The number of damaged tubules was reduced by the inhibition of MEG3 following IRI (Fig. 3H). Moreover, IRI induced mitophagy, and silencing of MEG3 inhibited mitophagy activity (Fig. 3I–K).

### MEG3 can bind with miR-145-5p in HK-2 cells following IR

**Fig. 4:**
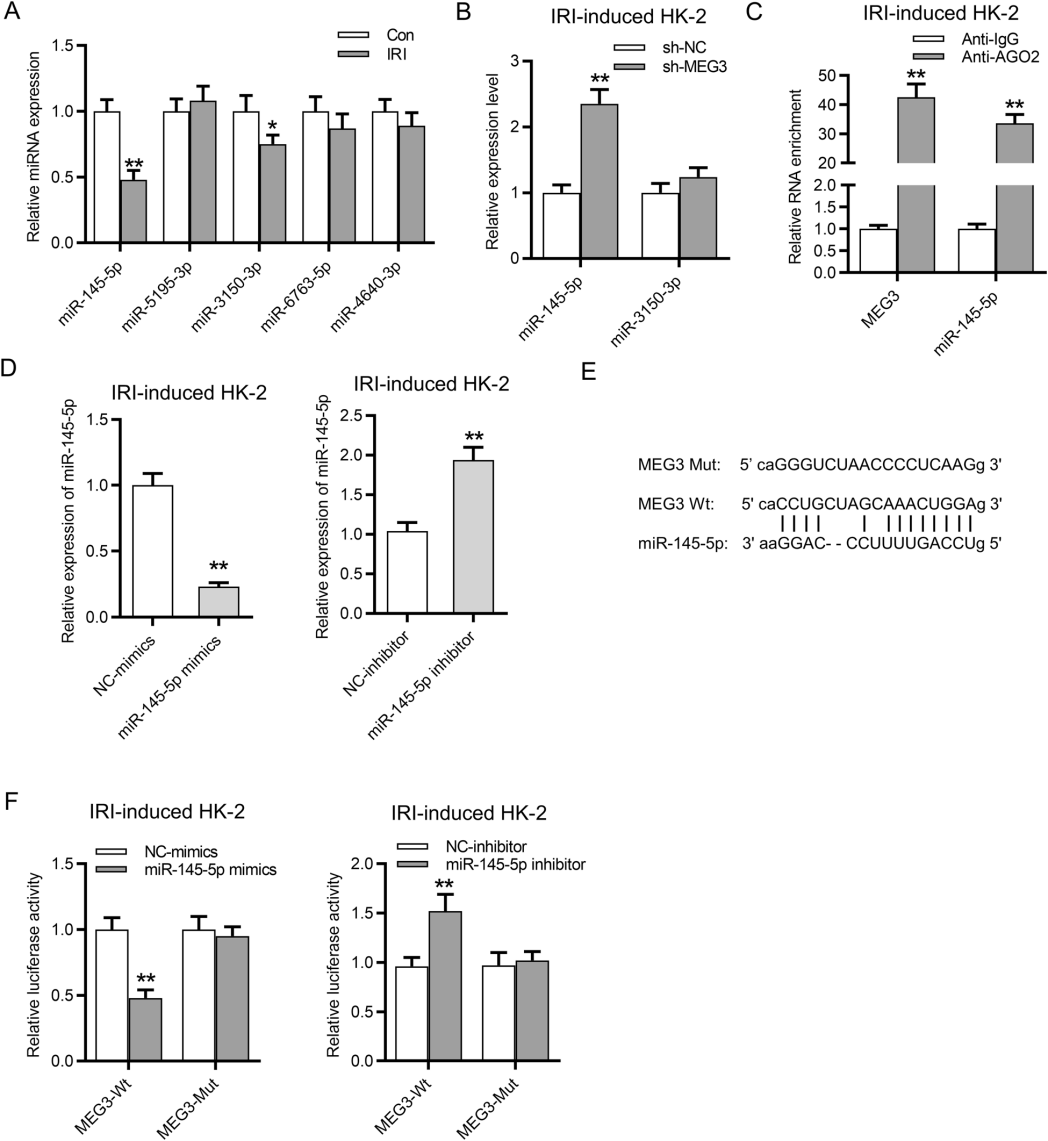
MEG3 can bind with miR-145-5p in HK-2 cells following IRI. **A** RT-qPCR evaluated the expression of five candidate miRNAs in HK-2 cells following IRI. **B** RT-qPCR identified the expression of miR-145-5p and miR-3150-3p in IR-treated HK-2 cells after transfection of sh-MEG3. **C** RIP assay was conducted to determine the relative enrichment of MEG3 and miR-145-5p in precipitations of anti-IgG and anti-Ago2 in HK-2 cells following IRI. **D** RT-qPCR revealed the overexpression and knockdown efficiency of miR-145-5p in HK-2 cells following IRI. **E** The binding site of MEG3 and miR-145-5p was predicted from starBase. **F** Luciferase reporter assay revealed the luciferase activity of pmirGLO-MEG3-Wt and pmirGLO-MEG3-Mut plasmids in HK-2 cells. *p < 0.05, **p < 0.01.

Afterwards, the underlying ceRNA mechanism of MEG3 involved in IRI was investigated. StarBase database (http://starbase.sysu.edu.cn/) was used to identify the potential miRNAs binding with MEG3 (screening condition: strict stringency (≥5) in CLIP data). According to the data, we found that there are five candidate miRNAs (miR-145-5p, miR-5195-3p, miR-3150-3p, miR-6763-5p, miR-4640-3p) that might bind with MEG3. Among them, miR-145-5p and miR-3150-3p were decreased in IR-treated HK-2 cells compared with that in the control group (Fig. 4A). The following assays were conducted in IR-treated HK-2 cells. Downregulation of MEG3 resulted in the increase of miR-145-5p expression and had no significant effect on miR-3150-3p in HK-2 cells (Fig. 4B). Thus miR-145-5p was applied for further molecular exploration. RNA immunoprecipitation (RIP) assay was subsequently conducted, and the results revealed that MEG3 and miR-145-5p were enriched in the Ago2 group rather than in the IgG group, indicating the coexistence of MEG3 and miR-145-5p in RNA-induced silencing complexes (Fig. 4C). Further, miR-145-5p was effectively overexpressed or knocked down in HK-2 cells by transfection of miR-145-5p mimics or miR-145-5p inhibitor with NC mimics or NC inhibitor as respective controls. Based on the data from RT-qPCR, the expression of miR-145-5p was increased by miR-145-5p mimics and was decreased by miR-145-5p inhibitor in IR-treated HK-2 cells (Fig. 4D). The binding sequences of miR-145-5p and MEG3 were predicted from starBase. We mutated the binding sequences of MEG3 on miR-145-5p to explore whether they are responsible for the binding between MEG3 and miR-145-5p (Fig. 4E). Luciferase reporter assay revealed that the luciferase activity of MEG3-WT was significantly reduced by miR-145-5p mimics and enhanced by miR-145-5p inhibitor, while the luciferase activity of MEG3-Mut presented no significant change in both groups (Fig. 4F).

### RTKN served as a target of miR-145-5p

**Fig. 5:**
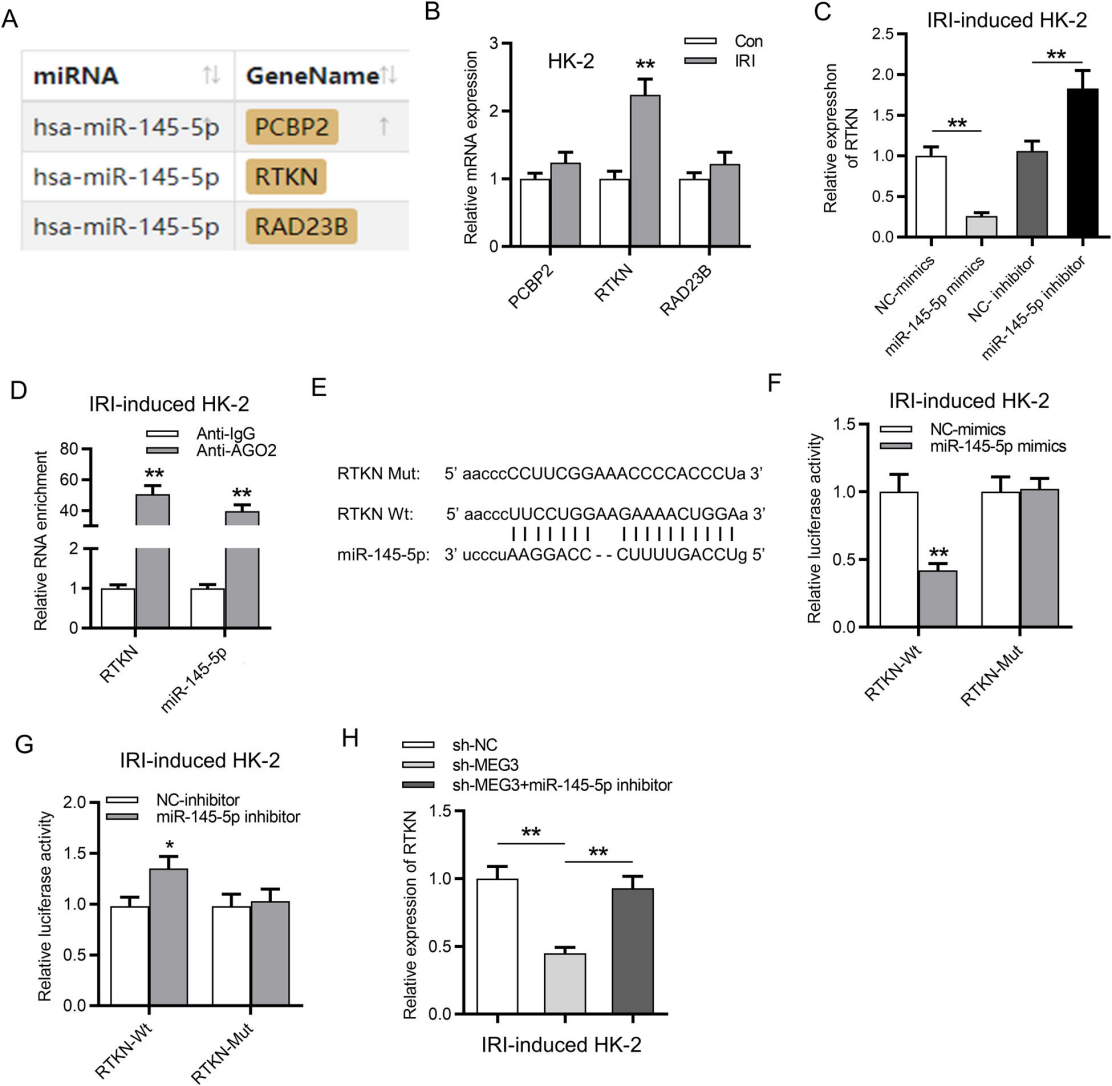
RTKN served as a target of miR-145-5p. **A** Three potential targets (PCBP2, RTKN, and RAD23B) for miR-145-5p were revealed. **B** Relative RTKN expression was determined in IR-treated HK-2 cells. **C** The influence of miR-145-5p mimics and miR-145-5p inhibitor on RTKN expression was determined by RT-qPCR. **D** RIP assay demonstrated relative enrichment of miR-145-5p and RTKN in the anti-IgG and anti-Ago2 groups. **E** The binding site of RTKN and miR-145-5p was predicted from starBase. **F**, **G** Luciferase activity revealed the influence of miR-145-5p mimics and miR-145-5p inhibitor on luciferase activity of pmirGLO RTKN-Wt and pmirGLO RTKN-Mut plasmids. **H** RTKN expression was determined by RT-qPCR in HK-2 cells transfected with sh-NC, sh-MEG3, and sh-MEG3 + miR-145-5p inhibitor. *p < 0.05, **p < 0.01.

Then the downstream targets of miR-145-5p were probed. As revealed in Fig. 5A, there are three potential targets (PCBP2, RTKN, and RAD23B) of miR-145-5p (screening condition: strict stringency (≥5) in CLIP data; high stringency (≥3) in degradome data; program number: 5 programs). RTKN expression was higher in HK-2 cells post IRI than in control cells, while the expression of PCBP2 and RAD23B exhibited no significant changes (Fig. 5B). Moreover, the mRNA expression of RTKN was increased by miR-145-5p inhibitor and was reduced by upregulation of miR-145-5p (Fig. 5C). Additionally, RIP assay demonstrated that miR-145-5p and RTKN were enriched in Ago2 group instead of IgG group (Fig. 5D), indicating that miR-145-5p and RTKN coexisted in RNA induced silenced complexes. Moreover, the predicted binding sites of miR-145-5p and RTKN were obtained from starBase (Fig. 5E). The upregulation of miR-145-5p decreased the luciferase activity of RTKN-WT, and downregulation of miR-145-5p increased the luciferase activity of RTKN-WT. However, both the transfection of miR-145-5p mimics and the miR-145-5p inhibitor exhibited no significant effects on the luciferase activity of RTKN-Mut, suggesting that miR-145-5p targeted RTKN at the predicted sites (Fig. 5F, G). Furthermore, silencing of MEG3 reduced the expression of RTKN, while inhibition of miR-145-5p rescued the suppressive effects of silenced MEG3 on RTKN expression, indicating that MEG3 upregulated RTKN expression via binding with miR-145-5p (Fig. 5H).

### Inhibition of RTKN reduced cell apoptosis and mitophagy in HK-2 cells followed by IRI

**Fig. 6:**
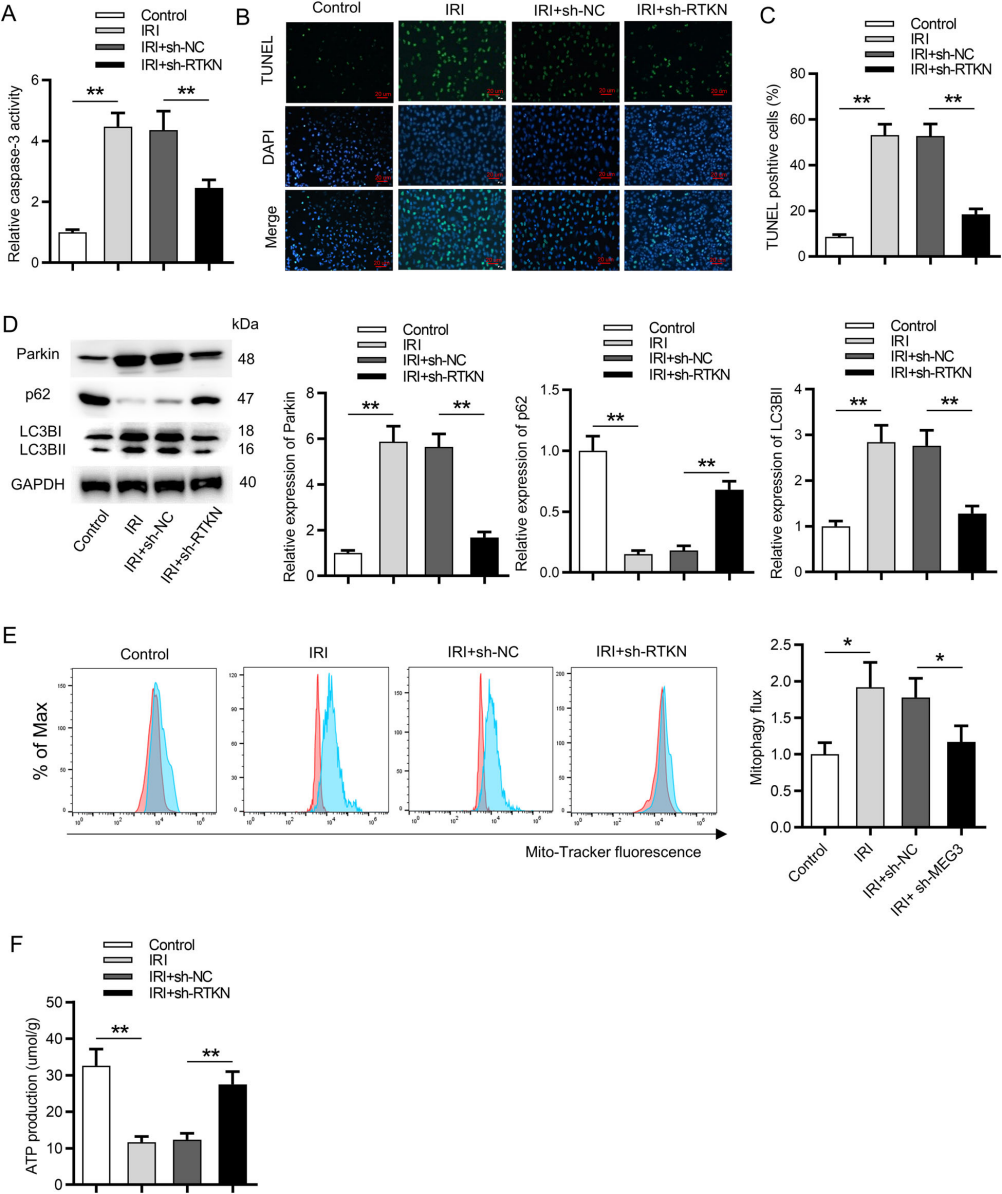
Inhibition of RTKN reduced cell apoptosis and mitophagy in HK-2 cells followed by IRI. **A** The influence of silenced RTKN on caspase-3 activity was determined via a caspase-3 activity kit. **B**, **C** A TUNEL assay detected the number of TUNEL-positive cells in IR-treated HK-2 cells transfected with sh-RTKN. **D** Western blot analysis showed the protein levels of Parkin, p62, LC3B-I, and LC3B-II in IRI-treated HK-2 cells transfected with sh-RTKN. **E** MitoTracker Green FM staining revealed the influence of silenced RTKN on mitophagy flux. Red color indicated DMSO and blue color indicated Baf. **F** Influence of silenced RTKN on ATP production in IR-treated HK-2 cells was measured. *p < 0.05, **p < 0.01.

Furthermore, the functions of RTKN on HK-2 cells following IRI were probed. We first silenced the expression of RTKN via transfection of sh-RTKN. The effective knockdown efficiency of RTKN was validated by RT-qPCR analysis (Fig. 6A). TUNEL assay depicted that silencing of RTKN rescued the promoting effect of IR treatment on cell apoptosis (Fig. 6B, C). The following function assays revealed that inhibition of RTKN partially restored mitophagy activity induced by IRI in HK-2 cells (Fig. 6D–F).

### Upregulation of RTKN rescued the effects of silenced MEG3 on cell apoptosis and mitophagy

**Fig. 7:**
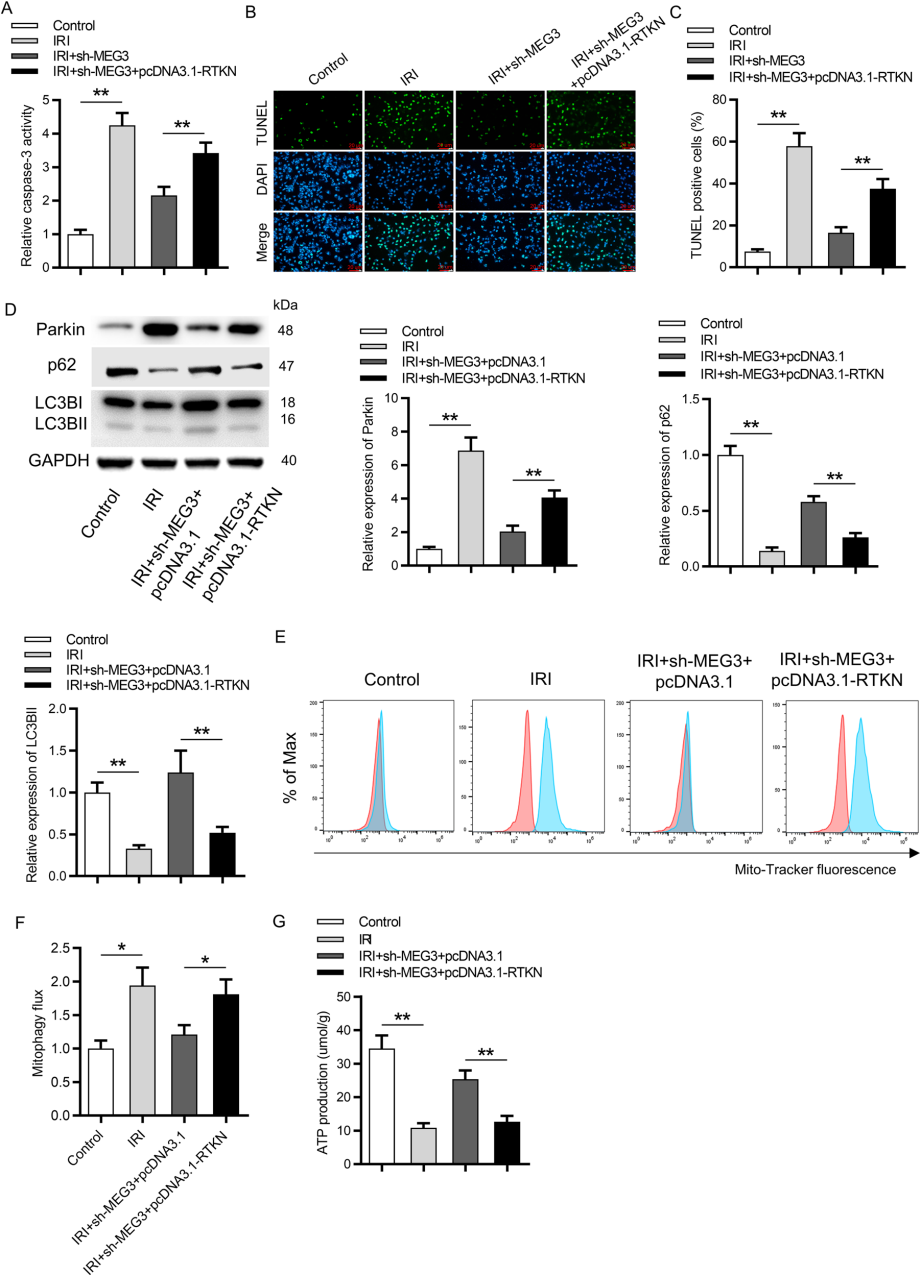
Upregulation of RTKN rescued the effect of silencing of MEG3 on cell apoptosis and mitophagy. **A** The rescue effect of upregulated RTKN on silenced MEG3 in caspase-3 activity was evaluated via a caspase-3 activity kit. **B**, **C** TUNEL assay revealed the rescue effect of upregulated RTKN on silenced MEG3 in the number of TUNEL-positive cells. **D** Western blot analysis showed the protein levels of Parkin, p62, LC3B-I, and LC3B-II in IR-treated HK-2 cells transfected with sh-MEG3 + pcDNA3.1-RTKN. **E**, **F** Mitophagy flux was assessed in IR-treated HK-2 cells after cotransfection with sh-MEG3 + pcDNA3.1-RTKN. Red color indicated DMSO and blue color indicated Baf. **G** ATP production in IRI-treated HK-2 cells cotransfected with sh-MEG3 + pcDNA3.1-RTKN was measured. *p < 0.05, **p < 0.01.

Subsequently, rescue assays were performed in IR-treated HK-2 cells. Suppression of MEG3 decreased the caspase-3 activity and RTKN overexpression rescued this effect (Fig. 7A). The number of TUNEL-positive cells was reduced in response to downregulation of MEG3 and was elevated by upregulation of RTKN (Fig. 7B, C). RTKN overexpression rescued the effects of inhibited MEG3 on the protein levels of Parkin, p62, LC3B-I, and LC3B-II (Fig. 7D). In addition, upregulation of RTKN rescued the inhibitory effects of silenced MEG3 on the mitophagy activity in IRI-treated HK-2 cells (Fig. 7E, F). In addition, increased ATP production caused by MEG3 knockdown was rescued by the overexpression of RTKN (Fig. 7G).

### c-MYC, the downstream effector of the Wnt/β-catenin pathway, promoted the transcription of MEG3

**Fig. 8:**
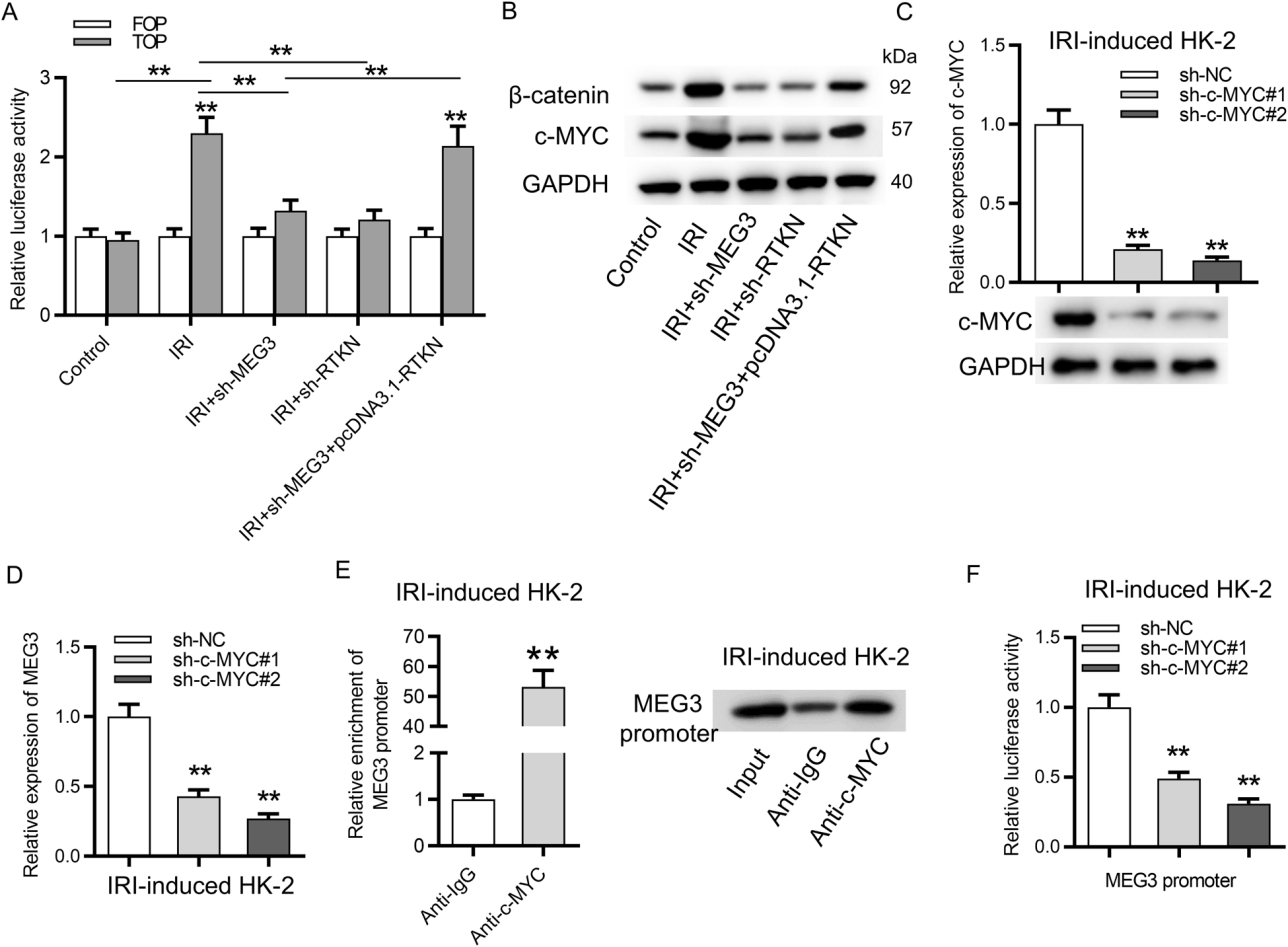
c-MYC, the downstream effector for the Wnt/β-catenin pathway, activated the transcription of MEG3. **A** FOP/TOP luciferase reporter assay revealed the influence of MEG3 and RTKN on the Wnt/β-catenin pathway in IR-treated HK-2 cells. **B** Western blot analysis of β-catenin and c-MYC proteins. **C** Knockdown efficiency of c-MYC was verified by RT-qPCR and western blot analyses. **D** The influence of silenced c-MYC on MEG3 expression was examined by RT-qPCR. **E** A ChIP assay was employed to detect relative enrichment of MEG3 promoter precipitated by anti-c-MYC. **F** A luciferase reporter assay was employed to detect the luciferase activity of MEG3 promoter in cells transfected with sh-c-MYC#1/2. **p < 0.01.

The Wnt/β-catenin pathway is activated in the pathogenesis of AKI. Both MEG3 and RTKN were reported to trigger the Wnt/β-catenin pathway. Thus we conducted the FOP/TOP luciferase reporter assay to detect the influence of MEG3 and RTKN on the Wnt/β-catenin pathway in IR-treated HK-2 cells. As revealed in Fig. 8A, the Wnt/β-catenin pathway was activated by IR treatment in HK-2 cells. Silencing of MEG3 or RTKN reduced the TOP activity, and upregulation of RTKN rescued the inhibitory effects of silenced MEG3 on TOP activity, indicating that MEG3 can activate the Wnt/β-catenin pathway by upregulation of RTKN. Moreover, protein expression of β-catenin and c-MYC was reduced by silenced MEG3 or RTKN, and RTKN rescued the inhibitory effects of silenced MEG3 on β-catenin and c-MYC protein levels (Fig. 8B). Furthermore, we identified that c-MYC is predicted to regulate MEG3^31^ and c-MYC commonly serves as a transcription factor. Whether transcription of MEG3 is activated by c-MYC was subsequently explored. mRNA and protein expression of c-MYC were reduced by transfection of sh-c-MYC#1/2 in IRI-treated HK-2 cells (Fig. 8C). Knockdown of c-MYC significantly reduced the expression of MEG3 (Fig. 8D). ChIP assay revealed that promoter of MEG3 was significantly precipitated by anti-c-MYC compared to anti-IgG (Fig. 8E). Finally, the luciferase reporter assay revealed that downregulation of c-MYC significantly reduced the luciferase activity of MEG3 promoter in IR-treated HK-2 cells (Fig. 8F). All these findings revealed that MEG3 activated the Wnt/β-catenin pathway by RTKN, and the downstream effector of the Wnt/β-catenin pathway, c-MYC, further promoted the transcription of MEG3 in HK-2 cells following IRI.

## Discussion

In the present study, MEG3 was upregulated in in vivo and in vitro models of IRI. Downregulation of MEG3 inhibited the apoptosis and mitophagy of HK-2 cells after IRI. As a cytoplasmatic lncRNA, MEG3 competitively bound with miR-145-5p to upregulate RTKN and thus activated the Wnt/β-catenin pathway. c-MYC, the downstream effector of the Wnt/β-catenin pathway, further activated MEG3 at the transcription level.

We first revealed the promoting effects of MEG3 on apoptosis and mitophagy of HK-2 cells post IRI. Another study has indicated that MEG3 inhibits autophagy to contribute to adenosine-induced cytotoxicity in hepatoma HepG2 cells^32^. MEG3 induces autophagy by upregulation of sirtuin 7 and inhibition on the PI3K/AKT/mTOR pathway in glioma cells^33^. MEG3 binds with miR-378 to inhibit the protective influence of miR-378 against neuronal autophagy and neurological functional impairment in ischemic stroke^34^. To explore the underlying ceRNA mechanism of MEG3 in HK-2 cells following IRI, the downstream miRNAs for MEG3 were investigated. MiR-145-5p was verified to bind with MEG3 and was negatively regulated by MEG3. It has been reported that activation of δ-opioid receptor modifies hypoxia-induced changes in expression of miR-145-5p in the liver^35^. MiR-145-5p induces the apoptosis of cardiomyocytes by DUSP6 via the c-Jun N-terminal kinase pathways^36^. Inhibition of miR-145-5p promotes neurological outcomes of rats post middle cerebral artery occlusion and reperfusion via the Nurr1-TNF-α signaling^37^. MiR-145-5p alleviates high glucose-induced apoptosis by regulating the Notch signaling pathway in podocytes^38^. In the current study, miR-145-5p expression was downregulated in IRI mice model and in HK-2 cells post IRI.

Subsequently, RTKN, a Rho-guanosine triphosphatase effector, was validated as the target of miR-145-5p. MiR-145-5p targeted the 3’-untranslated region of RTKN to inhibit the expression of RTKN. MEG3 can upregulate the expression of RTKN by competitively binding with miR-145-5p. The reports on role of RTKN in IRI are limited, and some studies have revealed the oncogenic functions of RTKN to inhibit cell apoptosis in colon cancer^39^, hepatocellular carcinoma^40^, lung cancer^41^, and breast cancer^42^. In the present study, we innovatively demonstrated that RTKN promoted apoptosis and mitophagy in HK-2 cells post IRI. Rescue assays further revealed that upregulation of RTKN rescued the effects of silenced MEG3 on apoptosis and mitophagy activity in IR-treated HK-2 cells.

The Wnt/β-catenin pathway is activated in AKI^43,44^ and can promote apoptosis of HK-2 cells^45,46^. The Wnt/β-catenin pathway can activate autophagy^47–49^ or be triggered by autophagy^50^. Moreover, MEG3 downregulates miR-183 to activate BRI3 and thus triggers the Wnt/β-Catenin pathway in BON1 cells^51^. MEG3 aggravates the hypoxia injury by Sox2 to activate the Wnt/β-catenin pathway in PC12 cells in a miR-147-dependent manner^52^. Additionally, RTKN2 has been indicated to activate the Wnt/β-catenin signaling pathway^53^. Our findings revealed that MEG3 can activate the Wnt/β-Catenin pathway by positive modulation on RTKN in a miR-145-5p-dependent manner. The pro-apoptotic functions of MEG3 on HK-2 cells might be dependent on the Wnt/β-Catenin pathway. c-MYC belong to the MYC family, is the downstream effector of the Wnt/β-catenin pathway, and was reported to regulate MEG3 expression^31^. In our study, we identified that c-MYC served as the transcription factor for MEG3 to activate upregulation of MEG3. Xiang et al. have revealed that MYC/c-MYC-induced cell proliferation serves as the main factor for mitochondrial decrease caused by mitophagy^54^. Xiong et al. have indicated that MYC impedes mitophagy-dependent necroptosis in diffuse large B cell lymphoma^55^. Another family member of MYC, n-MYC, blocks mitophagy and confers protection to reactive oxygen species in mitochondria via upregulation of TRAP1 in neuroblastoma^56^.

In conclusion, our study revealed that c-MYC-activated MEG3 aggravates IR-induced AKI through activating mitophagy and promoting apoptosis by upregulation of RTKN in a miR-145-5p-dependent way to trigger the Wnt/β-Catenin pathway, which might offer a new insight into the therapeutic methods for IR-induced AKI in the future.

## Materials and methods

### Ethics statement

The experimental protocol and animal use plan in this study were approved by the Animal Ethics Committee of Shengjing Hospital of China Medical University (Liaoning, China).

### IRI mice model

The kidney IRI mice models were established according to a previous study^57^. Eight-week-old adult C57BL/6 mice of both sexes weighing 22–25 g (*n* = 6–8/group) were obtained from Vital river (Beijing, China). Briefly, pentobarbital sodium was used for anesthetizing the mice by intraperitoneal injection (50 mg/kg). Post removing the hair, all mice were maintained on thermostatic blanket with a rectal probe to keep the body temperature at 36.5°C for surgery. The kidney pedicles were exposed and the flank incisions for bilateral clamping were conducted to block blood supply of kidney for inducing ischemia for 30 min followed by 12 h of reperfusion. Mice in the sham group received the same operation except clamping. Animals were grouped using a method of randomization. AAV-sh-MEG3 vector (serotype 2) was delivered (at the titer of 2.0 × 10^11^ v.g/mL) into mice via tail vein. The mice were sacrificed 28 days after AAV delivery. Finally, the kidneys were dissected, and blood was collected for biochemical assays.

### Cell culture and transfection

HK-2 cells (catalog number: CRL-2190) were obtained from American Type Culture Collection (ATCC) and were cultured in Dulbecco’s modified Eagle’s medium supplemented with 10% fetal bovine serum (Invitrogen, Carlsbad, CA, USA) at 37°C with 5% CO_2_ in a humidified atmosphere. Short hairpin RNA against MEG3 (sh-MEG3), miR-145-5p mimics/inhibitor, pcDNA3.1-RTKN vector, and respective controls (sh-NC, NC mimics/inhibitor, pcDNA3.1-NC) were constructed by GenePharma (Shanghai, China) and transfected into HK-2 cells using the Lipofectamine 2000 reagent (Invitrogen). Sequence for sh-MEG3 is GGACACATGAACGACTGAATT; sequence for miR-145-5p mimics is GUCCAGUUUUCCCAGGAAUCCCU; sequence for miR-145-5p inhibitor is AGGGAUUCCUGGGAAAACUGGAC; sequence for sh-c-MYC#1 is CAGTTGAAACACAAACTTGAA; and sequence for sh-c-MYC#2 is CCTGAGACAGATCAGCAACAA. At transfection for 48 h, cells were harvested for subsequent analyses.

### IRI cell model

HK-2 cells were used to construct the IRI cell model and were cultured in complete renal epithelial cell growth media with 95% air and 5% CO_2_ at 37°C. HK-2 cells were seeded onto a six-well plate. After 24 h of incubation, HK-2 cells were cultured with hypoxia treatment at 0.5% O_2_ for 15 h and were further cultured in normal culture medium with constant oxygen for 6 h.

### Determination of mitophagy

Mt-Keima is a fluorescent probe targeting the mitochondrial matrix sensitive to pH. The ratio of mt-Keima-derived fluorescence (543/458 nm) was calculated as the value of mitophagy. Low ratio mt-Keima emission (543/458 nm) reports a neutral environment, while high ratio fluorescence reports an acidic pH. Thus the difference between mitochondria in the cytoplasm and mitochondria in the acid lysosome can be distinguished by mt-Keima. Briefly, Mt-Keima probe was transfected into cells at 37°C in 5% CO_2_^58^. The fluorescence images were taken by Leica TCS SP5 II confocal spectroscopic microscope.

### Mitophagy flux

Mitophagy flux was evaluated as a previous study described. In brief, MitoTracker Green FM (500 nM) was used to stain cells. A CytoFlex platform (Beckman, South Kraemer Boulevard, Brea, CA, USA) was used for analysis. Mitophagy flux was defined as the ratio of MitoTracker Green FM fluorescence in the presence of mitophagy and lysosomal inhibitor (bafilomycin A1, Baf, 10 nM, Sigma-Aldrich, St. Louis, MO, USA) to that in the presence of only mitophagy.

### Determination of ATP levels

An ATP Assay Kit (Beyotime Institute of Biotechnology) was applied for determination of the levels of ATP in HK-2 cells. After reperfusion for 6 h, HK-2 cells were harvested and then lysed in lysis buffer followed by centrifugation at 12,000 × *g* for 10 min at 4°C. ATP levels were then detected via mixing the supernatant (50 mL) with luciferase reagent (50 mL). As luciferase catalyzes the oxidation of luciferin to produce light via employing ATP, thus the emitted light is linearly associated with the levels of the ATP. Therefore, the ATP levels were evaluated with a microplate luminometer (Varioskan Flash, 5250040, Thermo).

### Reverse transcription qPCR

Total RNA from kidney cortex and HK-2 cells was isolated with TRIzol reagent (Invitrogen). Then total RNA was reverse transcribed into cDNA with M-MLV Reverse Transcriptase (Invitrogen) and the miScript Reverse Transcription Kit (Qiagen, Germany) was used for miR-145-5p reverse transcription. With SYBR Premix Ex Taq™ II (Takara, Dalian, China), RT-qPCR was performed on a 7500 Fast RT-qPCR System. The 2^−ΔΔCT^ method was used to calculate the levels of MEG3, miR-145-5p, and RTKN, which were, respectively, normalized to U6 or glyceraldehyde 3-phosphate dehydrogenase (GAPDH). Primer sequences are listed in Supplementary Table.

### H&E staining

The kidney tissues of mice were harvested, fixed in formalin, embedded in paraffin, and dewaxed by xylene. Then kidney sections of 4 μm were subjected to hematoxylin and rinsed by distilled water. Furthermore, the tissues were stained by eosin, hydrated by gradient ethanol, cleared by xylene, air-dried, and mounted by neutral balsam. The optical microscope (Olympus BX53, Japan) was utilized for the determination of the pathological change of kidney injury after staining. Histologic changes, including tubular necrosis, tubular detachment, and tubule vacuole, were examined in a blinded manner. Renal tubules with loss of brush border, tubular dilation and disruption, cast formation, and cell lysis were considered damaged.

### Luciferase reporter assay

The wild-type or mutated sequence of MEG3 or RTKN was synthesized and subcloned into the pmirGLO-Dual-luciferase reporter vector (Promega, Madison, MI, USA) to construct the pmirGLO-MEG3-WT (wild type of MEG3), pmirGLO-MEG3-Mut (mutant-type of MEG3), pmirGLO-RTKN-WT (wild type of RTKN), and pmirGLO-RTKN-Mut (mutant-type of RTKN) plasmids, respectively. Mutant form of MEG3 was used to destabilize the binding between Mut-MEG3 and miR-145-5p. The pmirGLO-MEG3-WT or pmirGLO-MEG3-Mut (pmirGLO-RTKN-WT or pmirGLO-RTKN-Mut) containing the predicted binding sites of miR-145-5p were cotransfected with NC mimics or miR-145-5p mimics into HK-2 cells using Lipofectamine 2000. The c-MYC target sequences within MEG3 promoter were subcloned into the pGL3-Basis vector for promoter analysis to construct the pGL3-MEG3 promoter plasmids. The plasmids were cotransfected with sh-c-MYC#1/2 into the IR-treated HK-2 cells. After transfection for 48 h, the relative luciferase activity was detected with a Dual-luciferase Reporter Assay Kit (Promega), and the results were normalized to that of Renilla.

### Chromatin immunoprecipitation

An EZ ChIP Chromatin Immunoprecipitation Kit (Millipore, Billerica, MA) was applied for ChIP assay. Chromatin was cross-linked in 1% formaldehyde for 10 min at 37°C, sonicated to sizes of 200–1000 bp, followed by incubation with 3 μg of magnetic beads coated with anti-c-MYC and anti-IgG antibody (Millipore) overnight at 4°C for 2 h. Then protein/DNA complexes were eluted and reverse cross-linked. The immunoprecipitated DNA was analyzed by RT-qPCR.

### RNA immunoprecipitation

RIP assays were conducted using the Magna RIP RNA-Binding Protein Immunoprecipitation Kit (Millipore, MA, USA). HK-2 cells were lysed with the RIPA lysis buffer. Then 100 μL of cell extract was cultured with the RIPA buffer containing magnetic beads conjugated with human anti-Ago2 antibodies (Abcam, Cambridge, UK) or the IgG antibodies (Millipore, MA, USA). After antibody recovery by the protein A/G beads, the relative enrichment of MEG3, miR-145-5p, and RTKN was detected by RT-qPCR.

### Western blot analysis

RIPA lysate was used to extract proteins from renal tissues and HK-2 cells, and the concentrations were quantified by the Bicinchoninic Acid Kit (Pierce Biotechnology, Inc., Rockford, IL, USA). Sodium dodecyl sulfate–polyacrylamide gel electrophoresis was applied to isolate the proteins, and thereafter, separated proteins were transferred to polyvinylidene difluoride membranes (Millipore, Bradford, MA, USA). Membranes were blocked with 5% skimmed milk powder for 2 h and then cultured with primary antibodies against Parkin (ab77924; Abcam, Cambridge, UK), p62 (ab56416; Abcam), LC3B (ab192870; Abcam), and GAPDH (ab181602; Abcam) at 4°C overnight. Being washed twice with phosphate-buffered saline and Tween 20, the membranes were then cultured with horseradish peroxidase-labeled goat anti-rabbit secondary antibody for 1 h at 37°C. Enhanced chemiluminescence reagent (Millipore; Merck KGaA) was used to visualize the bands. The densitometric analyses of western blots were subjected to ImageJ program.

### Serum biochemical measurement

Serum creatinine and BUN levels in mice were measured by a commercially available clinical chemistry analyzer (Roche, Rotkreuz, Switzerland).

### Caspase-3 activity detection

The caspase-3 activity in HK-2 cells was assessed via the Caspase-3 Colorimetric Assay Kit (Beyotime, Shanghai, China). HK-2 cells were lysed by the lysis buffer. Then the supernatant of the lysate was gathered, centrifuged, and cultured in Ac-DEVE-pNA and reaction buffer at 37°C for 2 h. Finally, the optical density of caspase-3 activity at 405 nm was measured by a microplate reader (BioTek, VT).

### TUNEL assay

A TUENL assay was used to detect the TUNEL-positive cells in the kidney tissues. Briefly, tissue samples were fixed with formaldehyde and then dehydrated, paraffin embedded, and sectioned. Afterwards, the sections were dewaxed with xylene and hydrated with gradient ethanol. Thereafter, renal sections were cultured with 50 μL of TUNEL reaction solution for 50 min followed by converting agent peroxidase for 30 min at 37°C. Further, the sections were cultured with diaminobenzidine working solution for 10 min, counterstained with hematoxylin for 3 s, and sealed with neutral gum. Finally, the staining was observed under a microscope with five visual fields chosen from each group.

### Statistical analysis

Statistical analyses were conducted using the GraphPad Prism 5 software. Data from at least three independent experiments are presented as the mean ± standard deviation. Sample size was determined using G power for power analysis. All the data were normally distributed. Variance was similar between the groups that were being statistically compared. Student’s *t* test or one-way analysis of variance was performed to test differences between or among groups. *p* < 0.05 was considered statistically significant.

## Supplementary information

Supplementary Table
