# Supplementary material for: c-MYC-induced long noncoding RNA MEG3 aggravates kidney ischemia–reperfusion injury through activating mitophagy by upregulation of RTKN to trigger the Wnt/β-catenin pathway
Source: Cell Death Dis. 2021 Feb 18;12(2):191. doi: 10.1038/s41419-021-03466-5 (PMC7892540; doi:10.1038/s41419-021-03466-5)
Supplement: Supplementary file 1 — Supplementary Table [file 41419_2021_3466_MOESM1_ESM.docx]

**Supplementary Table Relative primer sequences**

| **Gene** | **Forward (5’-3’)** | **Reverse (5’-3’)** |
| --- | --- | --- |
| MEG3 (human) | GCCCTAGGGGAGTGACTACA | ACTCGGGACATACCTGCTCT |
| MEG3 (mice) | TGGGGATGGGTCTCTAGGTG | CCACTGACCCACAGTAACCC |
| miR-145-5p (human) | GTCCAGTTTTCCCAGGAATCCCT | GCTGTCAACATACGCTACGTAACG |
| miR-5195-3p (human) | TAGCAGACTCTTATGATG | TGGTGGAGTCGTCGTG |
| miR-3150-3p (human) | ACACTCCAGCTGGGCTGGGGAGATCCTCGA | TGGTGTCGTGGAGTCG |
| miR-6763-5p (human) | GGGATTCTGGAAGATGATGATGACCTGGGGAGTGG | GGACGGTAGCAAGCAAAGAGTGTG |
| miR-4640-3p (human) | GGGATTCTGGAAGATGATGATGACCACCCCCTGTT | GGACGGTAGCAAGCAAAGAGTGTG |
| PCBP2 (human) | AGGCAGGTTACCATCACTGG | CATTGTTCTAGCTGCTCCCC |
| RTKN (human) | GCCGCTGCTTACTATTGC | GTGCTTCCCGACTTTCTG |
| RAD23B (human) | ATGGCAACACTGGATAATGGC | TGTGAAGCAGCAACGATGAC |
| U6 (human) | CTCGCTTCGGCAGCACATA | AACGATTCACGAATTTGCGT |
| GAPDH (human) | GCAAGAGCACAAGAGGAAGA | ACTGTGAGGAGGGGAGATTC |
| GAPDH (mice) | TTAGCACCCCTGGCCAAGG | CTTACTCCTTGGAGGCCATG |
